# Controlling for Placebo Effects in Computerized Cognitive Training Studies With Healthy Older Adults From 2016-2018: Systematic Review

**DOI:** 10.2196/14030

**Published:** 2020-06-26

**Authors:** Alexander Masurovsky

**Affiliations:** 1 Berlin School of Mind and Brain Humboldt-Universität zu Berlin Berlin Germany

**Keywords:** computerized cognitive training, brain training, placebo, active control, elderly, older adults

## Abstract

**Background:**

Computerized cognitive training has been proposed as a potential solution to age-related cognitive decline. However, published findings from evaluation studies of cognitive training games, including metastudies and systematic reviews, provide evidence both for and against transferability from trained tasks to untrained cognitive ability. There continues to be no consensus on this issue from the scientific community. Some researchers have proposed that the number of results supporting the efficacy of cognitive training may be inflated due to placebo effects. It has been suggested that placebo effects need to be better controlled by using an active control and measuring participant expectations for improvement in outcome measures.

**Objective:**

This review examined placebo control methodology for recent evaluation studies of computerized cognitive training programs with older adult subjects, specifically looking for the use of an active control and measurement of expectations.

**Methods:**

Data were extracted from PubMed. Evaluation studies of computerized cognitive training with older adult subjects (age ≥50 years) published between 2016 and 2018 were included. Methods sections of studies were searched for (1) control type (active or passive) and subtype (active: active-ingredient or similar-form; passive: no-contact or passive-task); (2) if expectations were measured, how were they measured, and whether they were used in analysis; and (3) whether researchers acknowledged a lack of active control and lack of expectation measurement as limitations (where appropriate).

**Results:**

Of the 19 eligible studies, 4 (21%) measured expectations, and 9 (47%) included an active control condition, all of which were of the similar-form type. The majority of the studies (10/19, 53%) used only a passive control. Of the 9 studies that found results supporting the efficacy of cognitive training, 5 were for far transfer effects. Regarding the limitations, due to practical considerations, the search was limited to one source (PubMed) and to search results only. The search terms may have been too restrictive. Recruitment methods were not analyzed, although this aspect of research may play a critical role in systematically forming groups with different expectations for improvement. The population was limited to healthy older adults, while evaluation studies include other populations and cognitive training types, which may exhibit better or worse placebo control than the studies examined in this review.

**Conclusions:**

Poor placebo control was present in 47% (9/19) of the reviewed studies; however, the studies still published results supporting the effectiveness of cognitive training programs. Of these positive results, 5 were for far transfer effects, which form the basis for broad claims by cognitive training game makers about the scientific validity of their product. For a minimum level of placebo control, future evaluation studies should use a similar-form active control and administer a questionnaire to participants at the end of the training period about their own perceptions of improvement. Researchers are encouraged to think of more methods for the valid measure of expectations at other time points in the training.

## Introduction

### Cognitive Training as a Solution for Age-Related Cognitive Decline

As the many of the world’s nations face increasingly older populations, much attention has been given to how to limit the deleterious effects of aging on cognitive functioning, which are marked by a decrease in performance on a number of cognitive tests in the domains of memory, speed of processing, executive functioning, attention, and visual perception [1-4]. One proposed solution is computerized cognitive training, which has shown some promise in slowing age-related cognitive decline [5-7]. However, evaluation studies of computerized cognitive training have shown that it is still unclear whether success at training transfers to improved cognitive ability (see the following section for an explanation of transfer effects). Two opposing scientific consensus statements on this matter, signed by hundreds of scientists each, were published in 2014 [8,9]. Various studies have found significant effects of cognitive training on far transfer tests (indicating effects on broader categories of cognitive functioning) [5,6,10] or that cognitive training does little more than to improve abilities on near transfer tests (indicating effects on tasks similar to the training), with no indication of far transfer [11-13]. With such discrepancy in the field, some researchers have turned to analysis of the methodology used in cognitive training studies and identified, among other issues, a failure to properly control for placebo effects [12,14,15], which are systematic factors related to, but separate from, the training itself that may have been a causal component of observed effects [16] (see the “What Is the Placebo Effect” section for a more detailed explanation).

### Transfer Effects: Near and Far

The term “transfer effect” indicates a significant, positive change on an outcome measure that is separate from the training itself and observed after completion of the training. The level of ecological validity for a transfer effect is denoted by the preceding word “near,” “far,” and sometimes “real-world” or “daily life.” This review is concerned with claims of near and far transfer. If an outcome measure is very similar to the tasks in the training, this is considered a measure of near transfer. For example, a training game might have a gamified version of an n-back task, with slight variations; this would make an n-back test one of near transfer for this game. Other tests of specific elements of working memory might also be considered tests of near transfer. Far transfer is assessed by tests of broad cognitive domains, such as memory, processing speed, or cognitive control. More comprehensive tests of working memory or a conglomeration of tests of different working memory elements would be considered tests of far transfer. I am not aware of any official methods to determine whether a particular outcome measure is one of near or far transfer. Rather, the aforementioned general guidelines are used in this review to categorize studies as reporting near or far transfer in the event that they do not label their own findings as such. Any result indicating either near or far transfer effects will be referred to in this review as having a positive result.

### What Is the Placebo Effect?

The placebo effect has been studied for over 100 years [17] and is well-known in pharmaceutical research, especially research involving pain [18-20] and mood disorders [21-23]. In such studies, a sham treatment is administered in order to ensure that any positive response of the treatment is not due to the “symbols, rituals, and behaviors embedded in the clinical encounter” [24], such as the patient’s own expectations for improvement or social interactions with the clinician. Placebo effects do not necessarily replace an effect of the treatment; they can also occur alongside and interact with an effective treatment. For instance, a warm and caring demeanor on the part of the clinician administering medication may interact with the chemical components of the medication itself to produce an even stronger reduction in the patient’s subjective experience of pain [25]. The problem occurs when this effect systematically occurs differently in one experimental condition than in the other. If this phenomenon occurs without being detected by researchers, it could appear to be an effect caused by the experimentally manipulated variable (the medication). When properly controlled for, a control condition should elicit an equal placebo response to the experimental condition, strengthening evidence that any effect found was due to the treatment itself.

### Controlling for the Placebo Effect in Cognitive Training Studies: Active and Passive Controls

Pharmaceutical research uses double-blinding, random assignment, and utilization of a placebo treatment to minimize differences in possible placebo effects between control and experimental conditions. Two proposed solutions in cognitive training are the use of a carefully designed active control condition and the measurement of participant expectations for improvement [14]. In the following two sections, I give definitions of active controls and passive controls as well as two subtypes for each type of control that describe common methods for their implementation.

#### Active Controls: Active-Ingredient and Similar-Form Types

An active control game is meant to generate the same expectations for improvement on a cognitive assessment as the experimental training game, without providing the essential elements of the training that drive the effect on cognition. An ideal active control game, then, might be identical to the experimental game in every respect except for one, the hypothesized “active ingredient” that drives the effect of the game on cognitive performance [14,26]. For the purposes of this review, this will be referred to as the active-ingredient type of active control. In one example, in a multitasking game created to improve interference resolution (believed to be a component of executive control), Mishra et al [26] created a nearly identical game to serve as the active control but focused the challenge on the attention element of the game, rather than the interference element. In the second example, in a cognitive training game targeting multitasking ability, Anguera et al [27] created a single-tasking version of the game for the active control condition.

A similar-form type of active control mimics the training game in form but differs in details of the gameplay in more than one way. For instance, Cujek and Vranic [28] tested a computerized version of a challenging card game as cognitive training and used a computerized dice game as an active control. Between the conditions, the constants were the time spent playing a game at a computer and interaction with the experimenter. Ideally, participants would be unable to figure out from the gameplay if they were in the experimental or control condition, and expectations for improvement between conditions would remain constant. While perhaps less powerful than an active-ingredient type at generating similar expectations to the experimental training, the similar-form type is less demanding on study resources and is better suited to control for expectations than a passive control.

#### Passive Controls: No-Contact and Passive-Task Types

A common practice in cognitive training studies is to use a passive control game [12]. This review uses the term no-contact passive control to refer to studies where control subjects do not engage in any training and take only the pretest and posttest of cognition, often not speaking to researchers in between. This review uses the term passive-task passive control to refer to studies that have control subjects engage in an activity that shares little similarity with the experimental training program, such as watching educational DVDs or reading about cognitive training literature, and would be very unlikely to generate the same sorts of expectations (though expectation data could theoretically contradict this proposition).

### Evidence for Poor Placebo Control in Computerized Cognitive Training Studies

In a paper published in 2013, Boot et al [14] suggested that differences in expectations for improvement between groups could be responsible for some or all of the published positive results in the cognitive training literature. In several studies of the effects of video games on cognition [29,30], fast-paced action video games were tested as potential cognitive enhancers, with slower-paced video games as the control condition. Boot and colleagues [14] surveyed a sample uninvolved in other video game studies for general expectations regarding the video games tested in those studies. The results of the survey predicted the observed results found by those studies of video game play on cognition, supporting the claim by Boot et al [14] that placebo effects could not be ruled out as a driver of some or all of the observed effects. In 2016, a large-scale systematic review of cognitive training literature by Simons et al [12] concluded that, among other methodological issues, many studies still lacked an active control group and were therefore, at best, weak evidence for the effectiveness of the cognitive training games tested.

### Study Groups May Systematically Form Expectations That Impact Results

There is evidence that self-selection bias may be systematically contaminating results of cognitive training studies. Simply changing the method of recruitment created groups that would perform differently on outcome measures, despite being matched in nearly every other way. Overtly advertising the opportunity to participate in a cognitive training study (eg, “Brain Training & Cognitive Enhancement”) recruited a sample that achieved higher on tests of cognition after training, compared to a sample that was recruited via a neutral advertisement (eg, “Email Today & Participate in a Study”) [15]. Groups were matched for intelligence and motivation, and the groups performed equally well on the training tasks. The only measurable difference, other than performance on outcome measures, was that questionnaire data indicated the overtly recruited group had a stronger belief that intelligence is malleable rather than a fixed property of genetics. This study provides evidence that overt recruitment methods alone can create samples biased towards belief in the effectiveness of cognitive training, which can interact with the experimental condition to generate an improvement on outcome measures.

There are signs that overt methods are a common method of recruitment. Foroughi et al [15] checked recruitment methods for studies in a meta-analysis by another author [31] and found that 17 of the 19 studies had used overt recruitment methods. In this review, recruitment methods were not assessed; however, it is notable that the majority of studies may be unintentionally and systematically recruiting subjects that hold optimistic beliefs about the power of cognitive training.

### Other Evidence of Optimism About the Effectiveness of Cognitive Training

People generally believe that cognitive training is effective [32]. Furthermore, participants indicated higher or lower optimism for the effectiveness of cognitive training after the researchers showed them brief statements about the topic. The majority of elderly participants in one cognitive training study believed they had improved, even though no significant improvement had been found at the group level. Goghari and Lawlor-Savage [33] provided an analysis of expectation data; for actual cognitive training data, see the 2017 article by Goghari and Lawlor-Savage [34]. This indicates there may be a general optimism in elderly populations that cognitive training is effective.

### Placebo Effect Versus a Motivation Effect

There is another way to consider expectation effects: For cognitive training to be effective, a person needs to believe in it in order to approach the training in a motivated way. When is it a placebo effect, and when is it an interaction between the training program and motivation? I will differ on this question here. The question underlying this review was not the mechanism of how expectations may influence observed effects or even if expectations influence observed effects, but rather, in light of evidence that expectations may impact performance on outcome measures in cognitive training studies, are recent studies measuring and properly controlling for expectations between groups?

### Why Is it Imperative to Control for a Placebo Effect in Cognitive Training Research?

Unsupported claims about cognitive training carrying the blessing of scientific research can have direct consequences for society. Such claims may encourage people to spend time or money on cognitive training games, especially older adults that may be experiencing age-related cognitive decline and are therefore vulnerable to the marketing tactics of game makers. One example of such a claim about cognitive training studies is “What they show is that for the average person, our exercises truly speed up and sharpen the brain” [35]. Studies, such as that by Grönholm-Nyman et al [11], and meta-analyses, such as that by Sala and Gobet [36], have provided evidence against the existence of far transfer from cognitive training studies, but enough positive results exist [5,10] that the debate continues.

### Population: Healthy Older Adults

Cognitive ability may begin to decline due to age starting as early as 25 years old [37], resulting in a decrease in functionality for functions such as memory, speed of processing, executive functioning, attention, and visual perception [1-4]. Rather than viewing the new arrival of computerized cognitive training with fear or distrust, there are signs that older adults are generally optimistic about the effectiveness of such training [32,33]. There is some evidence that cognitive training may result in improvement on cognitive tests and effectively slow age-related cognitive decline in older adults [5-7], especially for narrow transfer effects [12,38]. However, there is an opportunity cost: If cognitive training is not effective at improving cognition or slowing decline, older adults could be wasting valuable time that could be spent exercising, socializing, or engaging in other activities for which there is stronger evidence of a benefit to cognition [12,39].

### Objective and Scope of This Review

In light of the diverging results in the cognitive training literature, evidence for potential widespread placebo effects in cognitive training studies should be taken seriously. If studies are not controlling for placebo effects, the possibility cannot be ruled out that this is a systematic methodological problem, falsely inflating the number of positive results. This review examined cognitive training literature from the previous 2 years (2016-2018) in studies of healthy older adults, specifically to determine the proportion of current studies focused on this population that properly controlled for potential placebo effects. Placebo control was assessed primarily by whether participant expectations for improvement were measured (in any form) and whether the study included an active control condition. Special attention was paid to studies that report far transfer effects. Studies with poor placebo control that find positive results for far transfer effects form the basis for the most extreme and potentially most egregious claims in the cognitive training game industry.

## Methods

### Registration

This review was not pre-registered. However, it was structured based on the PRISMA guidelines [40]. This review was conducted to fulfill a paper assignment for the Master’s Program at the Berlin School of Mind and Brain at Humboldt-Universität zu Berlin.

### Eligibility Criteria

Inclusion criteria were as follows: randomized controlled trials (including exploratory studies); participants ≥50 years old; participants not diagnosed with any cognitive disorder, including mild cognitive impairment; administration of the experimental cognitive training in electronic form, such as on a computer or tablet; outcomes were assessments of cognitive ability; and studies published between 2016 and 2018.

Exclusion criteria were as follows: training interventions of which the target was motor ability, such as gait; an experimental variable in a form other than that of computerized cognitive training, such as transcranial direct current stimulation (tDCS) dosage, exercise, or viewing instructional videos; reviews, metastudies, and other nonrandomized controlled trials; participants younger than the minimum age or with diagnosed cognitive impairment or neurodegenerative disease; studies published before 2016; and studies unavailable in an English translation.

The criteria were established to be able to make a generalization about current placebo control practices in studies that fit the aforementioned criteria. Motor activity as a target of cognitive training involves different enough sorts of training and tests than ones used for measurement of other cognitive abilities that it would widen the scope of the review too much. The time range was limited to the past 2 years so that the review can serve as a measurement of current standards of placebo control in cognitive training research. A healthy population was chosen because methods for recruitment, training, and testing of impaired populations may vary widely from those of healthy populations. Methodological practices in healthy populations of this age group may also be more easily comparable to those of healthy populations in other age groups. The definition of an older adult varies between studies, but the age was set to ≥50 years to be more inclusive.

### Information Sources

Studies were identified via a search of PubMed Online performed on December 1, 2018 by the study author. Each item that appeared in the search result was evaluated according to the inclusion and exclusion criteria.

### Search

The formula of search terms entered into PubMed was: (“2016/01/01”[Date - Publication] : “3000”[Date - Publication]) AND (cognitive[Title/Abstract]) AND (training[Title/Abstract]) AND ((game[Title/Abstract]) OR (computer[Title/Abstract])) AND ((older adult*[Title/Abstract]) OR (elderly[Title/Abstract])) NOT ((physical[Title]) OR (motor[Title/Abstract]) OR (exercise[Title]) OR (movement[Title]) OR (walking[Title/Abstract]) OR (gait[Title/Abstract]) OR (MCI[Title/Abstract]) OR (mild cognitive impairment[Title/Abstract]) OR (Alzheimer*[Title/Abstract])).

### Study Selection

Eligibility was assessed according to the previously described inclusion and exclusion criteria. Following the search, titles and abstracts of each returned result were reviewed for search criteria. If eligibility could not be determined in this way for a given paper, the methods section was inspected more closely. Eligibility was possible to determine for all studies using this method.

### Data Collection Process

The aforementioned search query was constructed by trial and error until most of the undesired study types (eg, motor ability as an outcome measure) were not returned in search results and many results for the desired study types (cognitive training game evaluations) were. A printout of results for the official search was saved. Each study on the list was then systematically checked for inclusion and exclusion criteria, first by looking at the title and abstract, then in the Methods section if needed. Data items of interest (see next section) were recorded in an Excel document for each included study.

### Data Items

The following data items were coded for each study. First, the measurement of expectations was coded as yes or no. If “yes” was determined for expectations measured, then how they were measured and if the expectation measurements were used in the analysis of the cognitive training results were recorded. Second, the control type (active or passive) was recorded. If the control type was active, the subtype (active-ingredient or similar-form) was recorded. If the control type was passive, the subtype (no-contact or passive-task) was recorded. Third, positive results were defined as a significantly higher score on the outcome measures for the experimental group. If positive results were found, the type (near transfer, far transfer, or both) was recorded. Fourth, if expectations were not measured, whether the authors mentioned this as a limitation in the discussion section (yes or no) was recorded. Fifth, if an active control was not used, whether the authors mentioned this as a limitation in the discussion section (yes or no) was recorded.

The reason for coding for positive results was to calculate the proportion of studies that found positive results that were performed without good placebo control (as defined by this review) compared to the overall number of included studies. This number was used as a metric of the proportion of cognitive training studies with methodology that may be biasing results and therefore, consensus in favor of efficacy.

Near and far transfer were determined based on whether the authors claimed their results reflected near or far transfer and on an investigation of the description of the outcome measure by the reviewer to see if these outcome measures were meant to reflect more general measures of cognition (far transfer) or specific tests of subcategories (near transfer).

Effect sizes were not recorded as this is review was meant as an inquiry into methodology, not as a synthesis of results.

### Summary Measures and Synthesis of Results

A summary of the data collected for the listed items is presented in the Results section. Conclusions were drawn about whether cognitive training studies are properly controlling for placebo, based on the proportion of studies measuring expectations and of those using an active control.

## Results

### Study Selection

The initial search of PubMed returned 38 results. Of these, 19 met the eligibility criteria, and 19 were excluded. Studies were excluded if: they were preregistered trials that had not yet returned results (3 articles), they were not evaluation studies of cognitive training on cognitive performance (15 articles), or were focused on subjects’ perceptions of the benefits of playing these games (1 article). The 15 articles excluded for being nonevaluation studies were comprised of: 2 correlational studies, 1 usability study, 1 survey of preferences (not expectations), 1 study with “flow” as an outcome measure (a measure of player absorption into a game), 1 evaluation study of tDCS on cognitive ability, 1 that used electroencephalogram gamma activity as an outcome measure, 1 that included a cohort of younger adults, and 6 studies that fell into one of the following categories: metastudy, review, conference proceedings, or opinion essay. Another paper was excluded because, although it stemmed from a cognitive training evaluation study, its focus was on subjects’ perceptions of the benefits of playing these games [33].

While it was not a study of a training game, 1 paper that was a study of general iPad training in improving cognition was included [41] because it was computerized training (via tablet) and followed the clinical evaluation format. Although it used several arcade-style games as the cognitive training and had other signs of methodological problems with reporting and possibly English translation issues (ie, used a dementia screening examination as a test of global cognition), 1 study [42] was included because it presented itself as an evaluation of “electronic cognitive training games” in its title; it was returned in the search results and had the potential to affect general perceptions of cognitive training game effectiveness.

### Study Characteristics

#### Studies That Measured Expectations

Of the 19 included studies, only 4 (21%) studies made any attempt to measure expectations to improve their experimental and control interventions (Table 1). Of those 4 studies, 3 used the expectation data in the interpretation of results. Of the 4 studies, 2 used an active control of the similar-form type, while 2 used a no-contact, passive control group (see next section for results on the use of active controls). Of the 4 studies that measured expectations, 2 found positive results for near transfer effects, and none found positive results for far transfer. Of the 15 studies that did not measure expectations, 5 mentioned this as a potential limitation of their study, while 10 did not, which suggests that the authors were either not aware of this as or did not believe this to be a potential confounding factor.

**Table 1:** Studies that measured expectations.

Search result #	Study	Control type	Control subtype	How were expectations measured?	Used in analysis of results?	Positive result?	Transfer
6	Souders et al (2017) [38]	Active	Similar-form	Expectation for improvement in various cognitive modalities measured at end of intervention	Yes	Yes	Near
14	Hynes (2016) [43]	Passive	No-contact	One question on questionnaire following training: “How much do you think you benefited from the training?” Rating: 2.62 of 4 (SD 0.67)	Yes	No	—^a^
21	Guye and von Bastian (2017) [44]	Active	Similar-form	Three questions after the post-assessment: improvement in trained tasks, in untrained tasks, and in everyday life tasks	Yes	No	—^a^
30	Yeo et al (2018) [45]	Passive	No-contact	One question after assessment: did your memory improve?	No	Yes	Near

^a^Positive results were not reported.

#### Active Control

Of the 19 included studies, 9 (47%) included an active control in the study design. None of these utilized an active-ingredient type game, such as that suggested by Mishra et al [46], where the control game mirrors the experimental in every way except for one factor hypothesized to drive the effect of training on cognitive ability. All 9 used a similar-form type of active control, where both groups play a game presented in a similar form for a similar amount of time, although details of the games themselves may vary (eg, the puzzle game suite, administered on a tablet, that served as the active control for the tablet-based training game suite used by Souders et al [38]). Of these studies, 3 also included a passive control group. Of the 9 studies using an active control, 7 found positive results: 6 for near transfer and 1 for far transfer.

#### Passive Control

Of the 19 included studies, 10 used a passive control and no active control group. Of these 10 studies, 5 used passive-task control groups, which completed simple tasks such as meeting the experimenter or reading information pamphlets about cognitive training. The other 5 studies used no-contact control groups that simply took the cognitive assessment tests before and after the experimental group had completed its training. Of the 10 studies that did not use an active control, 9 (90%) reported a significant positive effect of training, when compared with the control; 1 of these studies measured expectations but in a limited capacity and did not include this measurement in analysis of results. Of these 9 positive results, 5 were for near transfer effects, 2 were for far transfer effects, and 2 were for both near transfer and far transfer effects. Of the 10 studies that used only a passive control, only 4 mentioned this as a limitation to their study results, suggesting a potential lack of awareness on the part of the authors of the remaining 6 studies. Of these 6 studies that did not mention this limitation, 2 wrongly claimed to have included an “active placebo” condition (both studies had the same principle author; see the Discussion for more information).

#### Far Transfer

A significant, positive effect of training on tests of far transfer were reported by 6 studies. None of the studies measured expectations. Only 1 included an active control [28], 4 used passive-task controls, and 1 used a no-contact passive control.

### Results of Included Studies

Table 2 presents the results of the included studies.

**Table 2:** Results of included studies.

Search result #	Study	Control type	Control subtype	Description of conditions	Expectations measured?	Known limitation: expect-ations?	Known limitation: active control?	Positive result?	Transfer	Description of result
4	Cujzek & Vranic (2017) [28]	Active	Similar-form	Experimental: challenging computerized card game; active control: computerized dice-rolling game	No	No	—^a^	Yes	Far	Reasoning test (D-48) was different enough from skills required for the card game for authors to conclude that this was evidence of far transfer. Effect was maintained at 4-month follow up.
6	Souders et al (2017) [38]	Active	Similar-form	Experimental: Mind Frontiers suite; active control: puzzle game suite	Yes	—^b^	—^a^	Yes	Near	Corsi block tapping test (memory) was similar to game task. However, expectations for improvement may actually have MASKED observed improvement.
7	Kühn et al (2017) [47]	Both	Active: similar-form; passive: no-contact	Experimental: Inhibition training game on a tablet; active control: general-purpose cognitive training platform; passive: pretests and posttests only	No	No	—^a^	Yes	Near	Experimental group showed significant improvement on inhibition task while other groups did not.
8	Ballesteros et al (2017) [48]	Active	Similar-form	Experimental: Luminosity games; active control: The Sims or SimCity Build	No	No	—^a^	No	—^c^	Nonsignificant trend on n-back task for training; significant effect for group on oddball task in favor of active control
10	Pereira-Morales et al (2018) [49]	Passive	Passive-task	2 experimental groups: computerized training and computerized + pen and paper training; control: read brochure about cognitive training	No	No	No	Yes	Far	Experimental groups improved on cognitive tests more than passive control group. Experimental groups could not be considered active controls for each other because they were the same training, although one had additional training.
12	Lussier et al (2017) [50]	Passive	Passive-task	2 experimental groups: VPT^d^ vs FPT^e^; passive control: computer classes	No	No	No	Yes	Both	VPT had larger effect than FPT for near transfer and smaller effect for far transfer.
13	Toril et al (2016) [51]	Passive	Passive-task	Experimental: Luminosity, 15 1-hr training sessions; control: met with experimenter once a month	No	Yes	Yes	Yes	Both	Jigsaw puzzle task, digit forward (short-term memory), and Faces I and Faces II (episodic memory), Corsi Blocks; also maintained after 3 months (except for Corsi blocks)
14	Hynes (2016) [43]	Passive	No-contact	Experimental: watched videos about cognitive training and played adaptive online training games; control: took pretests and posttests	Yes	—^b^	No	No	—^c^	No difference found; therefore, no evidence of transfer effects (but: exploratory study)
15	Vaportzis et al (2017) [52]	Passive	No-contact	Experimental: learned how to use regular apps on a tablet; passive control: only took pretests and posttests	No	No	Yes	Yes	Near	Improved processing speed compared to control group
17	Lussier et al (2017) [53]	Passive	Passive-task	2 experimental conditions: heterogeneous training context and homogeneous training context; control: computer lessons	No	No	No	Yes	Near	Training groups both had significantly better scores on near transfer tests than control. Heterogeneous training led to steeper improvement of dual-task coordination learning curve.
18	Grönholm-Nyman et al (2017) [11]	Active	Similar-form	Experimental: task-switching training games; control: fun games (Tetris, Angry Birds, and Bejeweled)	No	Yes	—^a^	Yes	Near	Very limited: only one near-transfer effect found: overall accuracy on a rule-based part. Training group also improved on the training tasks (very near transfer).
21	Guye and von Bastian (2017) [44]	Active	Similar-form	Experimental: WM^f^ training games; control: visual search training games	Yes	—^b^	—^a^	No	—^c^	Bayesian analysis supported evidence for the null hypothesis. Expectation data went in opposite direction of observed result.
22	Chan et al (2016) [41]	Passive	Passive-task	Experimental: learned how to use a tablet; 2 control groups: games and radio programs at home, social groups met to discuss topics (to control for social interaction of training but limit new learning)	No	No	No	Yes	Far	iPad training improved processing speed and episodic memory compared with controls.
24	Perrot et al (2018) [54]	Both	Active: similar-for; passive: no-contact	Experimental: Kawashima Brain Training; active control: Super Mario Brothers; passive control: only pretests and posttests	No	Yes	—^a^	Yes	Near	Experimental training led to higher Stroop score than active control; both experimental training and active control had higher matrix reasoning scores than passive control; active control was significantly better at Corsi block test, spatial relations test, and number comparison test.
25	Nouchi et al (2016) [55]	Active	Similar-form	Experimental: processing-speed training game; active control: knowledge quiz training game	No	No	—^a^	Yes	Near	Improvements in processing speed, inhibition, and mood (depression scale) compared to active control
27	Belchior et al (2018) [56]	Both	Active control: similar-form; passive control: no-contact	Experimental: PositScience Insight (visual attention and processing speed); active control: Crazy Taxi; passive control: took pre and post-tests	No	Yes	—^a^	Yes	Near	Cognitive training improved visual attention and processing speed.
30	Yeo et al (2018) [45]	Passive	No-contact	Experimental: BrainFit software, which was controlled via BCI/dry EEG^g^ headband; passive control: took pretests and posttests	Yes	—^b^	Yes	Yes	Near	Men in intervention group outperformed men in control group on RBANS^h^ total score and subscore of Delayed Memory and Language.
31	Ordonez et al (2017) [42]	Passive	No-contact	Experimental: Actively Station cognitive training game suite, a series of games, many involving physical movement; passive control: took pretests and posttests	No	No	Yes	Yes	Far	Training group improved on global cognition, verbal fluency, memory complaints, and mood compared with control. Reported a significant result for language, which is contradicted by the data table, unless what was truly meant was verbal fluency. Other methodological problem: ACE-R^i^ is a dementia screening test.
34	Sosa and Lagana (2018) [57]	Passive	No-contact	Experimental: Brain Age; passive control: pretests and posttests	No	Yes	Yes	Yes	Near	Training group scored higher in brief syllable count and arithmetic assessments.

^a^The study included active control (not a limitation).

^b^The study controlled for expectations (not a limitation).

^c^The study reported no transfer effects.

^d^VPT: variable priority training.

^e^FPT: fixed priority training.

^f^WM: working memory.

^g^EEG: electroencephalogram.

^h^RBANS: Repeatable Battery for the Assessment of Neuropsychological Status.

^i^ACE-R: Addenbrooke's Cognitive Examination Revised.

### Synthesis of Results

The summary of the control for expectations is shown in Table 3. The first two columns report the proportion of included studies that took methodological steps identified in this review to control the influence of expectations: measuring expectations and including an active control condition. The third and fourth columns show the proportion of studies whose results may be misleading due to concerns raised in this review: those that did not include an active control, yet found a significant effect for their intervention, and those that additionally found a far transfer effect.

**Table 3:** Summary of expectation control (n=19).

Studies	Measured expectations, n (%)	Used active control (all similar-form), n (%)	No active control + positive result, n (%)	No active control + far transfer results, n (%)
Studies that fit the criteria	4 (21)	9 (47)	9 (47)	5 (26)

## Discussion

### Summary of Evidence

This review found that 4 of 19 studies (21%) measured expectations; therefore, the majority of studies (15/19, 79%) did not find it necessary to measure expectations for improvement. Of these 15 studies that did not measure expectations, 9 studies also did not mention this as a limitation of the study. A smaller majority of studies (10/19, 53%) did not include an active control condition, 6 of which did not mention this as a limitation. Perhaps most troubling is that 9 of 10 of the studies that did not include an active control also found a positive result; therefore, 9 of the 19 studies included here (47%) have published results reporting positive effects and did not properly control for placebo effects.

### Studies That Measured Expectations: Further Analysis

Expectations were measured in some form in 4 studies. Of these, 2 found positive results, 1 found null results, and 1 found evidence supporting the null hypothesis using Bayesian analysis.

Souders et al [38] provided the strongest evidence for a positive result that was not due to undetected placebo effects. They used an active control and administered a detailed expectation questionnaire. They found that the experimental group improved more than the active control group on the Corsi Block Tapping test, a test of memory similar to the training game task and therefore a near transfer result. The expectation data suggest that their subjects expected more improvement from the puzzle games (the active control) than from the intervention games, strengthening the claim that this was not a placebo effect. The authors suggest that expectation effects may even have gone in the opposite direction, potentially masking a bona fide effect from the training on cognition.

Guye and von Bastian [44] provide strong, expectation-supported data against the effectiveness of working memory training. Using Bayesian analysis, they found evidence in favor of the null hypothesis, suggesting that the computerized working memory training regimen they used had no greater effect than the active control condition on cognitive ability. To gather data on expectations, they asked whether their subjects believed they had improved in three areas: on training tasks, on untrained tests of cognition, and on real-life tasks. The expectation data suggest that participants believed that they had improved as a result of the training. The results of this study support other research [13,58], suggesting that working memory training in general may not confer any near or far transfer effects on cognition in healthy subjects.

In an exploratory study with a small sample, Hynes [43] found no effect for a computerized cognitive training program on a battery of cognitive tests, including a reasoning test correlated with fluid intelligence and a test of general intelligence. Responses to a question gauging participant expectations suggest that participants had moderate expectations for improvement, at least following the training period (mean 2.62/4, SD 0.67). The study used a no-contact passive control design, which provides weak control of potential placebo effects; however, it may strengthen a null result, under the assumption that expectations for improvement would be higher for the training group than the no-contact group. A potential rebuttal to this assumption is that the training program could discourage participants and lower expectations for improvement. In this case, however, participants indicated on a single expectation question, administered at the end of outcome testing, that they believed they had improved. More expectation questions could have potentially helped to better understand participant expectations.

Yeo et al [45] found a sex-dependent, near transfer effect for cognitive training on scores of the Repeatable Battery for the Assessment of Neuropsychological Status, which tests a number of cognitive domains including language, memory, attention, and visuospatial construction. Expectation data were collected in the form of one question presented after the outcome measures, asking participants if they believed their memory had improved as a result of the training; the data suggest that they did believe that it had. While the authors interpreted this as a subjective marker of cognitive improvement, it also could mean that their positive results are weakened by the expectation data, as they cannot rule out the possibility that positive expectations were responsible for the sex-dependent increase in performance. Their design used a no-contact control; thus, the expectation question was only administered to the cognitive training group. It cannot therefore be known whether expectations for improvement differed between conditions.

### False Definition of Active Control

Two studies, both with Lussier as the first author, claimed to include an “active placebo” condition [50,53]; however, an examination of the methods indicated that both studies used a passive control condition of the passive-task type, according to the definitions in this review. This distinction is important because a passive control provides weaker control for potential placebo effects than an active control. The training conditions in both studies were variations on group-administered, computer-based cognitive training; the control condition in both studies was computer classes, teaching how to use software such as Microsoft Excel. While the participants engaged in learning in both conditions, expectations for improvement on cognitive assessments could be very different for a training program that exists for the purpose of improving cognition, rather than to simply teach particular skills. Expectations were not measured, so there is no reason to believe that expectations for improvement were held constant across conditions. There is an argument to be made that the two experimental conditions in each study served as active control conditions for each other; however, the authors did not present nor analyze their data in this way [50,53].

### Recommendations for Researchers of Cognitive Training Games

Measuring expectations can add to the practical workload of conducting a study. However, as demonstrated by some of the studies in this review, there are ways to measure expectations without significantly adding to the burden of data collection. All studies that collected expectation data that were reviewed here did so in the form of a questionnaire administered after the outcome measures. While potentially problematic because it cannot indicate what expectations were before or during training, it provides at least some insight into what expectations subjects held by the end of the training. This post hoc expectation data, while limited, can still be used in analysis of the observed results from the outcome tests. The study by Souders et al [38] serves as a model of expected data collection using a variety of expectation questions at the end of the training (note: Walter R. Boot, of Boot et al [14], was an author on this study and presented analysis of expectations towards the experimental and control games in a separate paper [59]). This study is also commendable for its use of an active control, of the similar-form type. Their experimental design strongly supports their claim that observed results were not likely to be placebo effects.

Using a variety of expectation questions can help elucidate whether participants formed expectations for improvement in specific cognitive modalities. Two of the studies reviewed here [43,45] used only one question to assess expectations and accordingly lacked a detailed understanding of participant expectations. The study by Hynes [43] was exploratory, so a fully placebo-controlled design may not have been practically feasible; however, it serves as an example of how simply administering more questions could help to clarify participant expectations. The mean score for responses to this study’s expectation question implied participants thought they had improved due to the training; further questions could clarify whether they found the training discouraging, for instance, or if they believed they had improved on one specific modality, such as memory, but not another. The study by Yeo et al [45] could have also benefited from additional expectation questions, as their expectation data reveal very limited information about participant expectations.

An active control provides all of the same benefits as a passive control, plus other benefits, such as expectation control. Beyond practical feasibility, there is no compelling methodological reason to forego use of an active control in favor of a passive control. No-contact passive controls offer some insight into the effects of repeated testing, but no control for any other placebo-related factors [16]. Passive-task control conditions can control for social interaction effects, but likely do not control for expectation effects, as participants can likely determine if they are in the control or training condition and form expectations for improvement accordingly.

More work can be done to find ways to measure expectations, both for the conditions used in specific studies and for general expectations regarding various types of cognitive training. Boot et al [14] used a separate sample to measure expectations; Rabipour et al [32] surveyed people about expectations for cognitive training in general. Foroughi et al [15] examined the effects of recruitment methods on performance after training and measured belief in the ability to improve one’s own intelligence. There are, perhaps, creative new ways in which researchers could assess expectations before and during cognitive training or to gain insight into other placebo-related factors [16], such as social interaction, that may be impacting performance.

### Limitations

This review was necessarily limited in its scope: Only one researcher was available, and it was conducted as one assignment to fill a paper requirement for a Master’s program. Only one search site was used (PubMed), the Works Cited sections of included papers were not screened for potential additions that matched the review criteria, and this review was not preregistered.

The search terms may have been too restrictive by only returning results that contained the terms “game” or “computer” in the title or abstract. One study of cognitive training, by Goghari and Lawlor-Savage [34] was not found by this search query. This study measured expectations and reported a null result. It would have slightly changed the results of this review by increasing the number of studies that attempted to measure expectations from 4/19 to 5/20.

Recruitment methods were not assessed in this review. Recruitment methods may play an important role in controlling for placebo effects [15] and would be a good consideration for future methodological reviews.

Other types of studies that fell outside the scope of this review should be reviewed for placebo control methodology, as they are also potentially subject to placebo effects. Studies involving populations of unhealthy older adults, for instance, are numerous and should be considered; cognitive training may or may not be an effective way to mitigate degeneration and related symptoms due to neurodegenerative disorders such as Alzheimer’s disease or even for less severe conditions such as mild cognitive impairment. Combined studies of neurostimulation, such as tDCS, with cognitive training may yield effects not present with tDCS or cognitive training alone. Such studies form a recent area of cognitive training research and should be examined for placebo-control methodology as well.

### Conclusions

In summary, this review found that the majority of computerized cognitive training studies with older adult samples from the past 2 years, which were included in this systematic review, have not measured expectations or properly controlled for potential placebo effects by including an active control. Methodologically speaking, only one study here [28] found placebo-controlled evidence for far transfer effects of cognitive training, although some that used an active control found evidence for near transfer effects. Studies that did measure expectations did so by administering an expectation questionnaire at the end of the training period. Cognitive training evaluation studies should, at a minimum, include a similar-form active control and measure expectations at the end of the training period. Ideally, future evaluation studies will include an active-ingredient type of active control and find creative new ways to measure expectations and control for other unintended types of placebo effects.

If cognitive training can truly be effective, scientists have just as much of a duty to find evidence for this possibility as they do to find evidence against it. Well-controlled studies that rule out placebo as the reason for any observed effects strengthen the result, whether as evidence for or against the effectiveness of cognitive training.

## Abbreviations

ACE-R: Addenbrooke's Cognitive Examination Revised
EEG: electroencephalogram
FPT: fixed priority training
RBANS: Repeatable Battery for the Assessment of Neuropsychological Status
tDCS: transcranial direct current stimulation
VPT: variable priority training
WM: working memory
